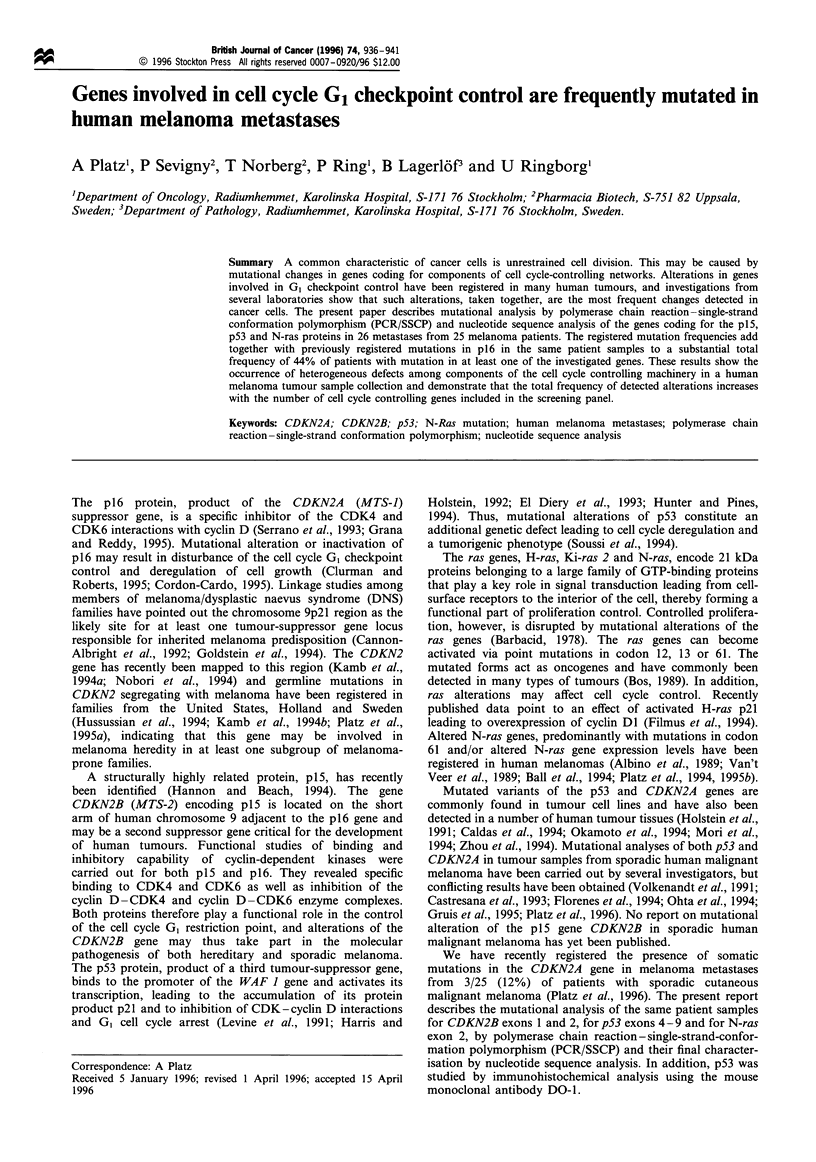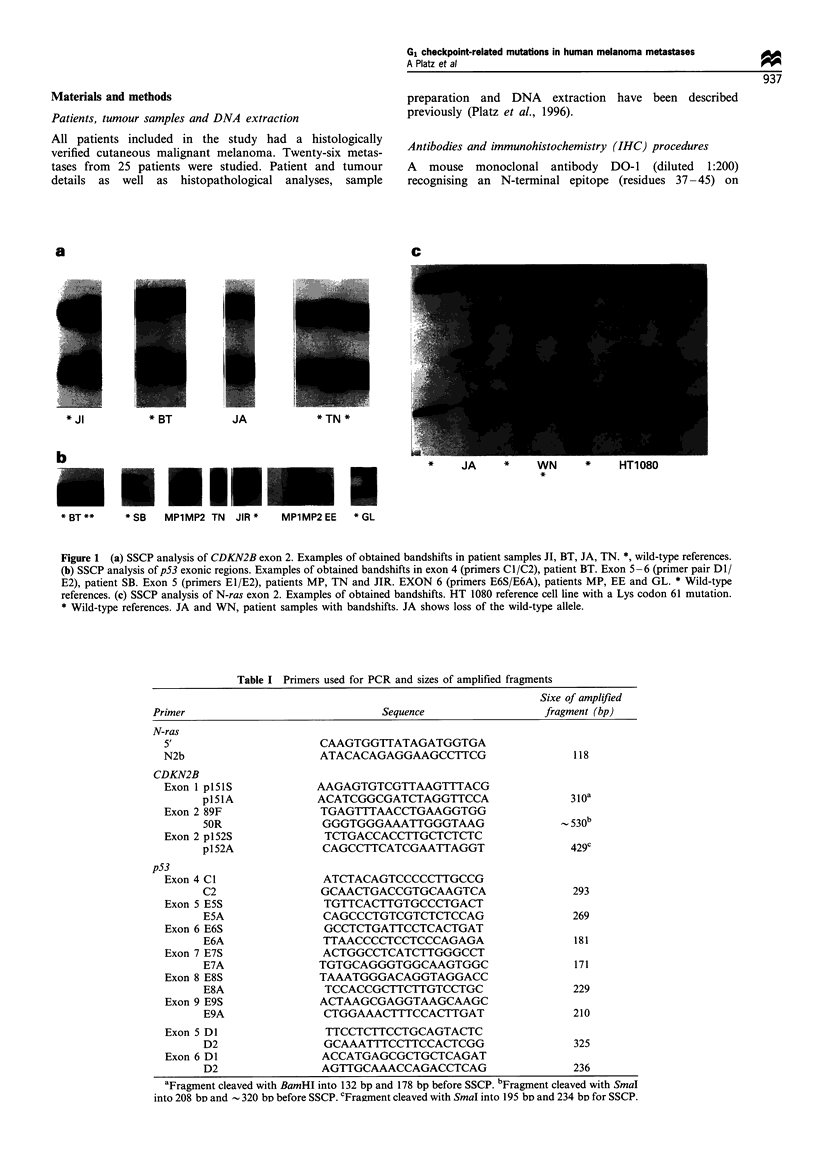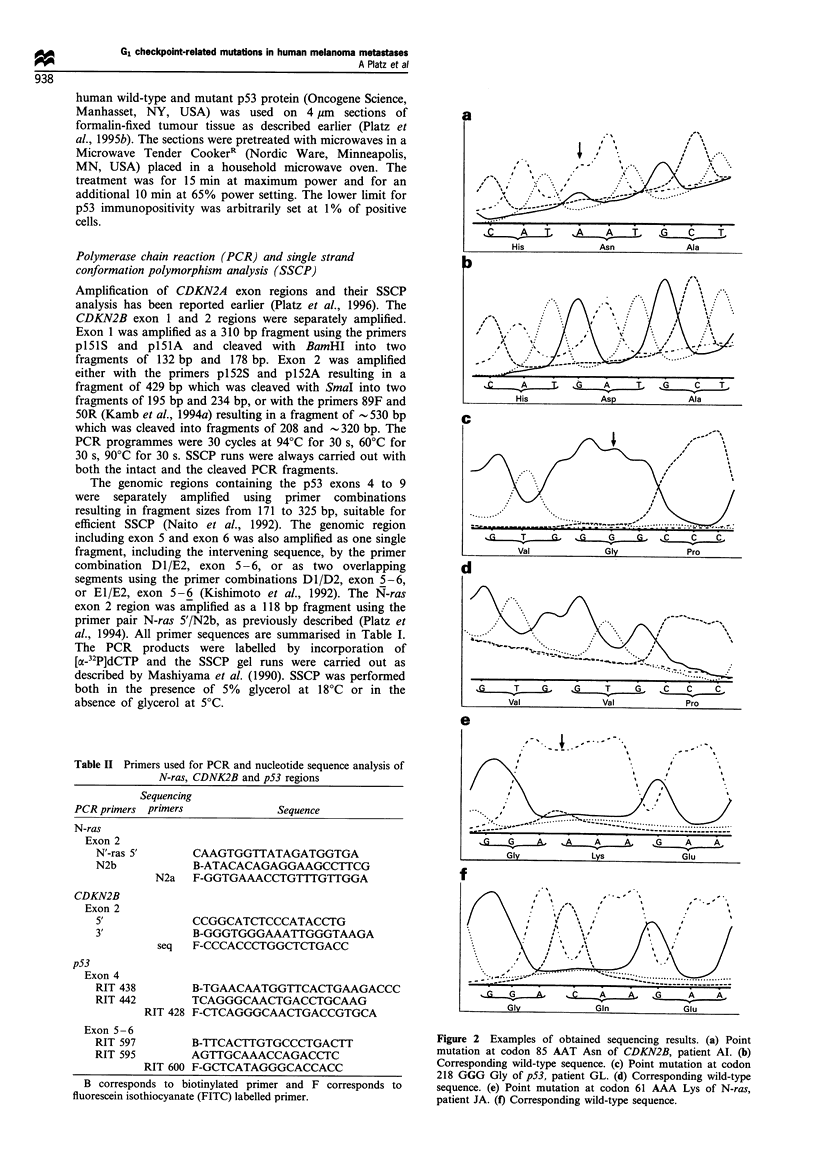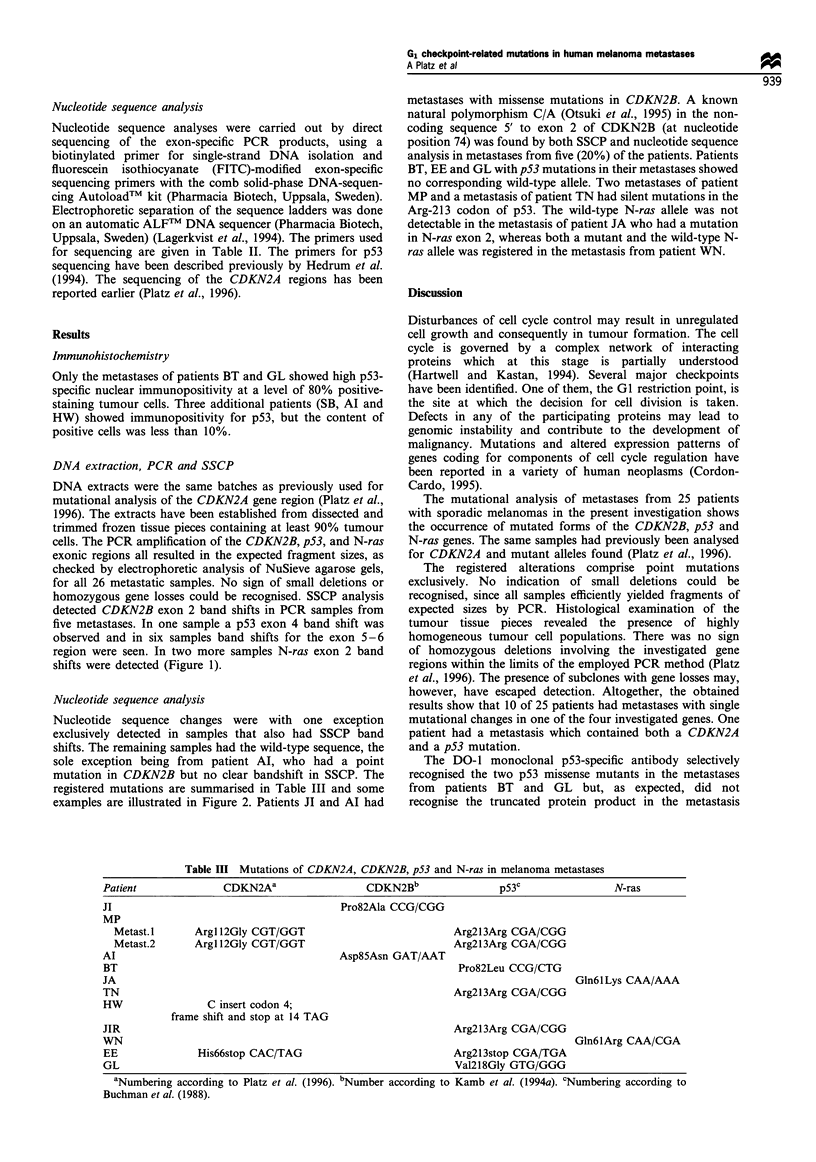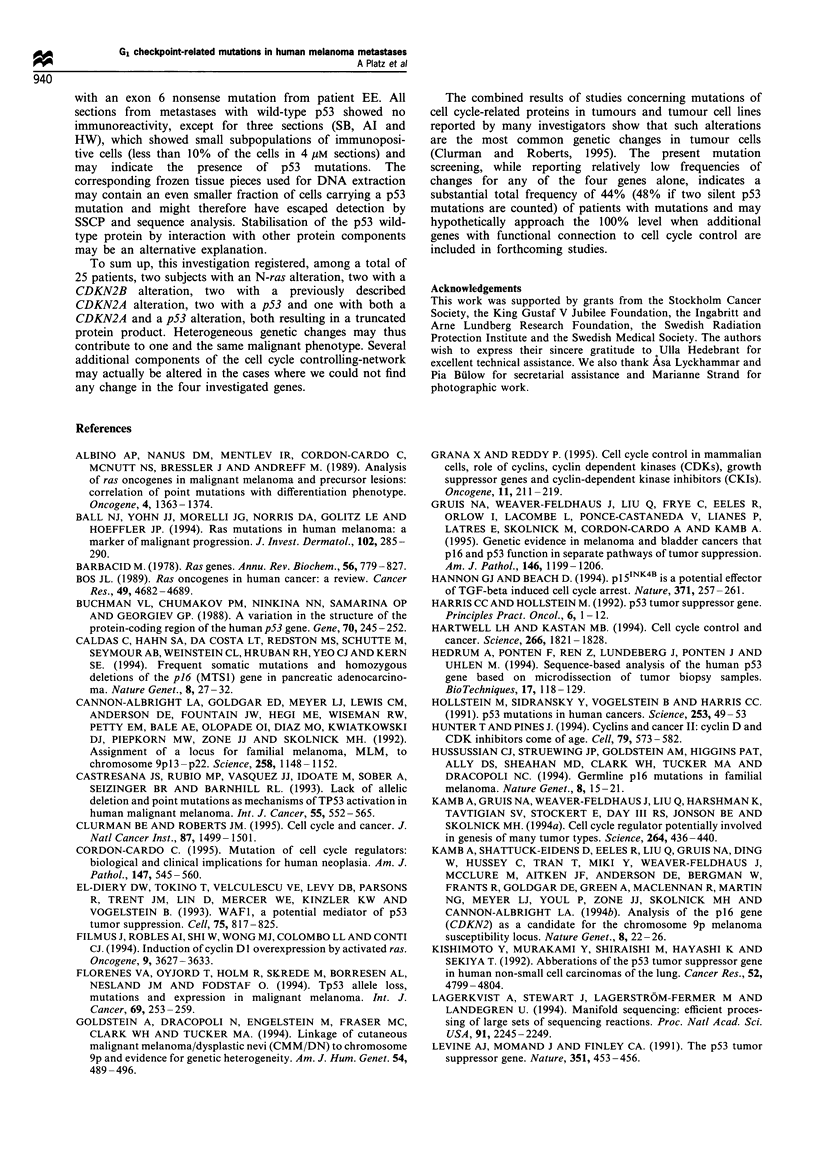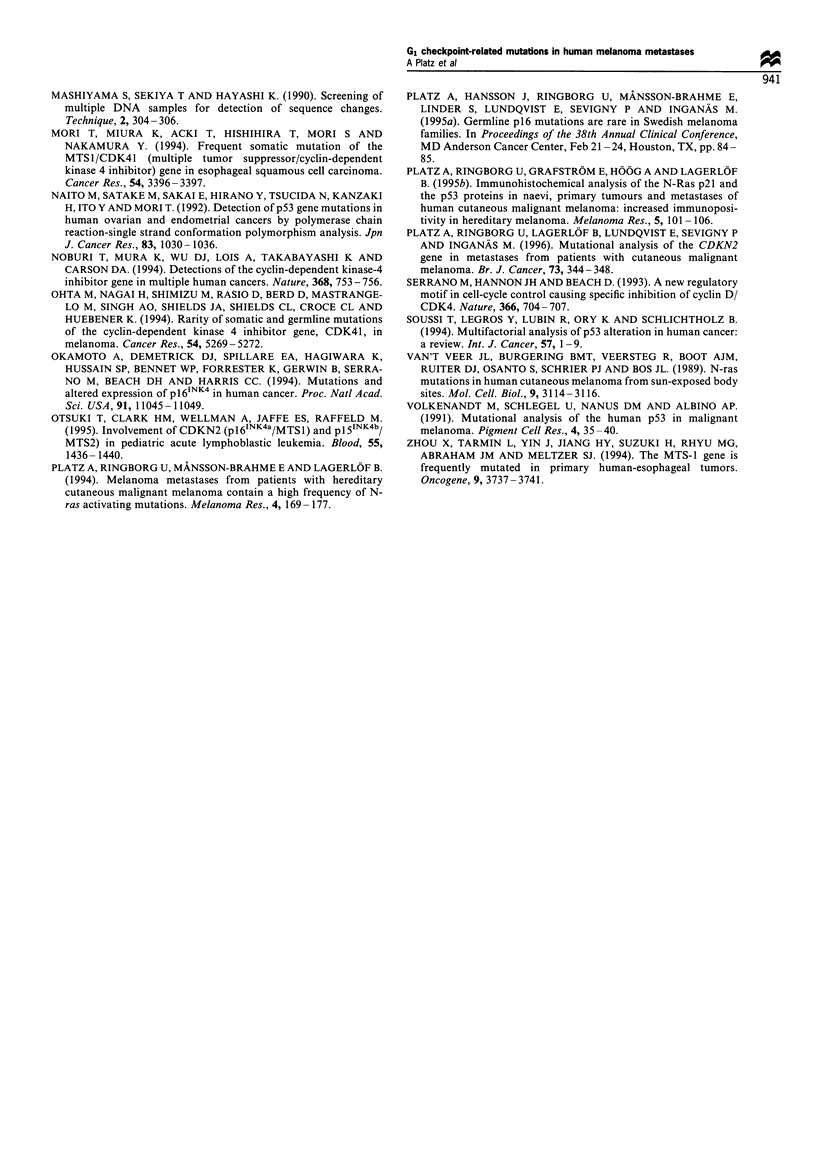# Genes involved in cell cycle G1 checkpoint control are frequently mutated in human melanoma metastases.

**DOI:** 10.1038/bjc.1996.460

**Published:** 1996-09

**Authors:** A. Platz, P. Sevigny, T. Norberg, P. Ring, B. Lagerlöf, U. Ringborg

**Affiliations:** Department of Oncology, Radiumhemmet, Karolinska Hospital, Stockholm, Sweden.

## Abstract

**Images:**


British Journal of Cancer (1996) 74, 936-941
© 1996 Stockton Press All rights reserved 0007-0920/96 $12.00

### Genes involved in cell cycle $G_1$ checkpoint control are frequently mutated in human melanoma metastases

A Platz[1], P Sevigny[2], T Norberg[2], P Ring[1], B Lagerlöf[3] and U Ringborg[1]

[1]*Department of Oncology, Radiumhemmet, Karolinska Hospital, S-171 76 Stockholm;* [2]*Pharmacia Biotech, S-751 82 Uppsala, Sweden;* [3]*Department of Pathology, Radiumhemmet, Karolinska Hospital, S-171 76 Stockholm, Sweden.*

**Summary** A common characteristic of cancer cells is unrestrained cell division. This may be caused by mutational changes in genes coding for components of cell cycle-controlling networks. Alterations in genes involved in $G_1$ checkpoint control have been registered in many human tumours, and investigations from several laboratories show that such alterations, taken together, are the most frequent changes detected in cancer cells. The present paper describes mutational analysis by polymerase chain reaction-single-strand conformation polymorphism (PCR/SSCP) and nucleotide sequence analysis of the genes coding for the p15, p53 and N-ras proteins in 26 metastases from 25 melanoma patients. The registered mutation frequencies add together with previously registered mutations in p16 in the same patient samples to a substantial total frequency of 44% of patients with mutation in at least one of the investigated genes. These results show the occurrence of heterogeneous defects among components of the cell cycle controlling machinery in a human melanoma tumour sample collection and demonstrate that the total frequency of detected alterations increases with the number of cell cycle controlling genes included in the screening panel.

**Keywords:** *CDKN2A; CDKN2B; p53;* N-*Ras* mutation; human melanoma metastases; polymerase chain reaction-single-strand conformation polymorphism; nucleotide sequence analysis

The p16 protein, product of the *CDKN2A* (*MTS-1*) suppressor gene, is a specific inhibitor of the CDK4 and CDK6 interactions with cyclin D (Serrano *et al.*, 1993; Grana and Reddy, 1995). Mutational alteration or inactivation of p16 may result in disturbance of the cell cycle $G_1$ checkpoint control and deregulation of cell growth (Clurman and Roberts, 1995; Cordon-Cardo, 1995). Linkage studies among members of melanoma/dysplastic naevus syndrome (DNS) families have pointed out the chromosome 9p21 region as the likely site for at least one tumour-suppressor gene locus responsible for inherited melanoma predisposition (Cannon-Albright *et al.*, 1992; Goldstein *et al.*, 1994). The *CDKN2* gene has recently been mapped to this region (Kamb *et al.*, 1994*a*; Nobori *et al.*, 1994) and germline mutations in *CDKN2* segregating with melanoma have been registered in families from the United States, Holland and Sweden (Hussussian *et al.*, 1994; Kamb *et al.*, 1994*b*; Platz *et al.*, 1995*a*), indicating that this gene may be involved in melanoma heredity in at least one subgroup of melanoma-prone families.

A structurally highly related protein, p15, has recently been identified (Hannon and Beach, 1994). The gene *CDKN2B* (*MTS-2*) encoding p15 is located on the short arm of human chromosome 9 adjacent to the p16 gene and may be a second suppressor gene critical for the development of human tumours. Functional studies of binding and inhibitory capability of cyclin-dependent kinases were carried out for both p15 and p16. They revealed specific binding to CDK4 and CDK6 as well as inhibition of the cyclin D-CDK4 and cyclin D-CDK6 enzyme complexes. Both proteins therefore play a functional role in the control of the cell cycle $G_1$ restriction point, and alterations of the *CDKN2B* gene may thus take part in the molecular pathogenesis of both hereditary and sporadic melanoma. The p53 protein, product of a third tumour-suppressor gene, binds to the promoter of the *WAF 1* gene and activates its transcription, leading to the accumulation of its protein product p21 and to inhibition of CDK-cyclin D interactions and $G_1$ cell cycle arrest (Levine *et al.*, 1991; Harris and Holstein, 1992; El Diery *et al.*, 1993; Hunter and Pines, 1994). Thus, mutational alterations of p53 constitute an additional genetic defect leading to cell cycle deregulation and a tumorigenic phenotype (Soussi *et al.*, 1994).

The *ras* genes, H-*ras*, Ki-*ras 2* and N-*ras*, encode 21 kDa proteins belonging to a large family of GTP-binding proteins that play a key role in signal transduction leading from cell-surface receptors to the interior of the cell, thereby forming a functional part of proliferation control. Controlled proliferation, however, is disrupted by mutational alterations of the *ras* genes (Barbacid, 1978). The *ras* genes can become activated via point mutations in codon 12, 13 or 61. The mutated forms act as oncogenes and have commonly been detected in many types of tumours (Bos, 1989). In addition, *ras* alterations may affect cell cycle control. Recently published data point to an effect of activated H-*ras* p21 leading to overexpression of cyclin D1 (Filmus *et al.*, 1994). Altered N-*ras* genes, predominantly with mutations in codon 61 and/or altered N-*ras* gene expression levels have been registered in human melanomas (Albino *et al.*, 1989; Van't Veer *et al.*, 1989; Ball *et al.*, 1994; Platz *et al.*, 1994, 1995*b*).

Mutated variants of the p53 and *CDKN2A* genes are commonly found in tumour cell lines and have also been detected in a number of human tumour tissues (Holstein *et al.*, 1991; Caldas *et al.*, 1994; Okamoto *et al.*, 1994; Mori *et al.*, 1994; Zhou *et al.*, 1994). Mutational analyses of both *p53* and *CDKN2A* in tumour samples from sporadic human malignant melanoma have been carried out by several investigators, but conflicting results have been obtained (Volkenandt *et al.*, 1991; Castresana *et al.*, 1993; Florenes *et al.*, 1994; Ohta *et al.*, 1994; Gruis *et al.*, 1995; Platz *et al.*, 1996). No report on mutational alteration of the p15 gene *CDKN2B* in sporadic human malignant melanoma has yet been published.

We have recently registered the presence of somatic mutations in the *CDKN2A* gene in melanoma metastases from 3/25 (12%) of patients with sporadic cutaneous malignant melanoma (Platz *et al.*, 1996). The present report describes the mutational analysis of the same patient samples for *CDKN2B* exons 1 and 2, for *p53* exons 4-9 and for N-*ras* exon 2, by polymerase chain reaction-single-strand-conformation polymorphism (PCR/SSCP) and their final characterisation by nucleotide sequence analysis. In addition, p53 was studied by immunohistochemical analysis using the mouse monoclonal antibody DO-1.

Correspondence: A Platz
Received 5 January 1996; revised 1 April 1996; accepted 15 April 1996

### Materials and methods

*Patients, tumour samples and DNA extraction*

All patients included in the study had a histologically verified cutaneous malignant melanoma. Twenty-six metastases from 25 patients were studied. Patient and tumour details as well as histopathological analyses, sample preparation and DNA extraction have been described previously (Platz *et al.*, 1996).

*Antibodies and immunohistochemistry (IHC) procedures*

A mouse monoclonal antibody DO-1 (diluted 1:200) recognising an N-terminal epitope (residues 37–45) on

**Figure 1** (**a**) SSCP analysis of *CDKN2B* exon 2. Examples of obtained bandshifts in patient samples JI, BT, JA, TN. *, wild-type references. (**b**) SSCP analysis of *p53* exonic regions. Examples of obtained bandshifts in exon 4 (primers C1/C2), patient BT. Exon 5–6 (primer pair D1/E2), patient SB. Exon 5 (primers E1/E2), patients MP, TN and JIR. EXON 6 (primers E6S/E6A), patients MP, EE and GL. * Wild-type references. (**c**) SSCP analysis of N-*ras* exon 2. Examples of obtained bandshifts. HT 1080 reference cell line with a Lys codon 61 mutation. * Wild-type references. JA and WN, patient samples with bandshifts. JA shows loss of the wild-type allele.

**Table I** Primers used for PCR and sizes of amplified fragments

| *Primer* | *Sequence* | *Sixe of amplified fragment (bp)* |
|---|---|---|
| *N-ras* | | |
| 5′ | CAAGTGGTTATAGATGGTGA | |
| N2b | ATACACAGAGGAAGCCTTCG | 118 |
| *CDKN2B* | | |
| Exon 1 p151S | AAGAGTGTCGTTAAGTTTACG | |
| p151A | ACATCGGCGATCTAGGTTCCA | 310[a] |
| Exon 2 89F | TGAGTTTAACCTGAAGGTGG | |
| 50R | GGGTGGGAAATTGGGTAAG | ~530[b] |
| Exon 2 p152S | TCTGACCACCTTGCTCTCTC | |
| p152A | CAGCCTTCATCGAATTAGGT | 429[c] |
| *p53* | | |
| Exon 4 C1 | ATCTACAGTCCCCCTTGCCG | |
| C2 | GCAACTGACCGTGCAAGTCA | 293 |
| Exon 5 E5S | TGTTCACTTGTGCCCTGACT | |
| E5A | CAGCCCTGTCGTCTCTCCAG | 269 |
| Exon 6 E6S | GCCTCTGATTCCTCACTGAT | |
| E6A | TTAACCCCTCCTCCCAGAGA | 181 |
| Exon 7 E7S | ACTGGCCTCATCTTGGGCCT | |
| E7A | TGTGCAGGGTGGCAAGTGGC | 171 |
| Exon 8 E8S | TAAATGGGACAGGTAGGACC | |
| E8A | TCCACCGCTTCTTGTCCTGC | 229 |
| Exon 9 E9S | ACTAAGCGAGGTAAGCAAGC | |
| E9A | CTGGAAACTTTCCACTTGAT | 210 |
| Exon 5 D1 | TTCCTCTTCCTGCAGTACTC | |
| D2 | GCAAATTTCCTTCCACTCGG | 325 |
| Exon 6 D1 | ACCATGAGCGCTGCTCAGAT | |
| D2 | AGTTGCAAACCAGACCTCAG | 236 |

[a]Fragment cleaved with *Bam*HI into 132 bp and 178 bp before SSCP. [b]Fragment cleaved with *Sma*I into 208 bp and ~320 bp before SSCP. [c]Fragment cleaved with *Sma*I into 195 bp and 234 bp for SSCP.

human wild-type and mutant p53 protein (Oncogene Science, Manhasset, NY, USA) was used on 4 μm sections of formalin-fixed tumour tissue as described earlier (Platz *et al*., 1995*b*). The sections were pretreated with microwaves in a Microwave Tender Cooker[R] (Nordic Ware, Minneapolis, MN, USA) placed in a household microwave oven. The treatment was for 15 min at maximum power and for an additional 10 min at 65% power setting. The lower limit for p53 immunopositivity was arbitrarily set at 1% of positive cells.

*Polymerase chain reaction (PCR) and single strand conformation polymorphism analysis (SSCP)*

Amplification of *CDKN2A* exon regions and their SSCP analysis has been reported earlier (Platz *et al*., 1996). The *CDKN2B* exon 1 and 2 regions were separately amplified. Exon 1 was amplified as a 310 bp fragment using the primers p151S and p151A and cleaved with *Bam*HI into two fragments of 132 bp and 178 bp. Exon 2 was amplified either with the primers p152S and p152A resulting in a fragment of 429 bp which was cleaved with *Sma*I into two fragments of 195 bp and 234 bp, or with the primers 89F and 50R (Kamb *et al*., 1994*a*) resulting in a fragment of ~530 bp which was cleaved into fragments of 208 and ~320 bp. The PCR programmes were 30 cycles at 94°C for 30 s, 60°C for 30 s, 90°C for 30 s. SSCP runs were always carried out with both the intact and the cleaved PCR fragments.

The genomic regions containing the p53 exons 4 to 9 were separately amplified using primer combinations resulting in fragment sizes from 171 to 325 bp, suitable for efficient SSCP (Naito *et al*., 1992). The genomic region including exon 5 and exon 6 was also amplified as one single fragment, including the intervening sequence, by the primer combination D1/E2, exon 5–6, or as two overlapping segments using the primer combinations D1/D2, exon 5–6, or E1/E2, exon 5–6 (Kishimoto *et al*., 1992). The N-*ras* exon 2 region was amplified as a 118 bp fragment using the primer pair N-*ras* 5′/N2b, as previously described (Platz *et al*., 1994). All primer sequences are summarised in Table I. The PCR products were labelled by incorporation of [α-$^{32}$P]dCTP and the SSCP gel runs were carried out as described by Mashiyama *et al*. (1990). SSCP was performed both in the presence of 5% glycerol at 18°C or in the absence of glycerol at 5°C.

**Table II** Primers used for PCR and nucleotide sequence analysis of *N-ras*, *CDNK2B* and *p53* regions

| *PCR primers* | *Sequencing primers* | *Sequence* |
|---|---|---|
| N-*ras* | | |
| Exon 2 | | |
| N′-ras 5′ | | CAAGTGGTTATAGATGGTGA |
| N2b | | B-ATACACAGAGGAAGCCTTCG |
| | N2a | F-GGTGAAACCTGTTTGTTGGA |
| *CDKN2B* | | |
| Exon 2 | | |
| 5′ | | CCGGCATCTCCCATACCTG |
| 3′ | | B-GGGTGGGAAATTGGGTAAGA |
| | seq | F-CCCACCCTGGCTCTGACC |
| *p53* | | |
| Exon 4 | | |
| RIT 438 | | B-TGAACAATGGTTCACTGAAGACCC |
| RIT 442 | | TCAGGGCAACTGACCTGCAAG |
| | RIT 428 | F-CTCAGGGCAACTGACCGTGCA |
| Exon 5–6 | | |
| RIT 597 | | B-TTCACTTGTGCCCTGACTT |
| RIT 595 | | AGTTGCAAACCAGACCTC |
| | RIT 600 | F-GCTCATAGGGCACCACC |

B corresponds to biotinylated primer and F corresponds to fluorescein isothiocyanate (FITC) labelled primer.

**Figure 2** Examples of obtained sequencing results. (**a**) Point mutation at codon 85 AAT Asn of *CDKN2B*, patient AI. (**b**) Corresponding wild-type sequence. (**c**) Point mutation at codon 218 GGG Gly of *p53*, patient GL. (**d**) Corresponding wild-type sequence. (**e**) Point mutation at codon 61 AAA Lys of N-*ras*, patient JA. (**f**) Corresponding wild-type sequence.

*Nucleotide sequence analysis*

Nucleotide sequence analyses were carried out by direct sequencing of the exon-specific PCR products, using a biotinylated primer for single-strand DNA isolation and fluorescein isothiocyanate (FITC)-modified exon-specific sequencing primers with the comb solid-phase DNA-sequencing Autoload™ kit (Pharmacia Biotech, Uppsala, Sweden). Electrophoretic separation of the sequence ladders was done on an automatic ALF™ DNA sequencer (Pharmacia Biotech, Uppsala, Sweden) (Lagerkvist *et al.*, 1994). The primers used for sequencing are given in Table II. The primers for p53 sequencing have been described previously by Hedrum *et al.* (1994). The sequencing of the *CDKN2A* regions has been reported earlier (Platz *et al.*, 1996).

### Results

*Immunohistochemistry*

Only the metastases of patients BT and GL showed high p53-specific nuclear immunopositivity at a level of 80% positive-staining tumour cells. Three additional patients (SB, AI and HW) showed immunopositivity for p53, but the content of positive cells was less than 10%.

*DNA extraction, PCR and SSCP*

DNA extracts were the same batches as previously used for mutational analysis of the *CDKN2A* gene region (Platz *et al.*, 1996). The extracts have been established from dissected and trimmed frozen tissue pieces containing at least 90% tumour cells. The PCR amplification of the *CDKN2B*, *p53*, and N-*ras* exonic regions all resulted in the expected fragment sizes, as checked by electrophoretic analysis of NuSieve agarose gels, for all 26 metastatic samples. No sign of small deletions or homozygous gene losses could be recognised. SSCP analysis detected *CDKN2B* exon 2 band shifts in PCR samples from five metastases. In one sample a p53 exon 4 band shift was observed and in six samples band shifts for the exon 5–6 region were seen. In two more samples N-*ras* exon 2 band shifts were detected (Figure 1).

*Nucleotide sequence analysis*

Nucleotide sequence changes were with one exception exclusively detected in samples that also had SSCP band shifts. The remaining samples had the wild-type sequence, the sole exception being from patient AI, who had a point mutation in *CDKN2B* but no clear bandshift in SSCP. The registered mutations are summarised in Table III and some examples are illustrated in Figure 2. Patients JI and AI had metastases with missense mutations in *CDKN2B*. A known natural polymorphism C/A (Otsuki *et al.*, 1995) in the non-coding sequence 5′ to exon 2 of CDKN2B (at nucleotide position 74) was found by both SSCP and nucleotide sequence analysis in metastases from five (20%) of the patients. Patients BT, EE and GL with *p53* mutations in their metastases showed no corresponding wild-type allele. Two metastases of patient MP and a metastasis of patient TN had silent mutations in the Arg-213 codon of p53. The wild-type N-*ras* allele was not detectable in the metastasis of patient JA who had a mutation in N-*ras* exon 2, whereas both a mutant and the wild-type N-*ras* allele was registered in the metastasis from patient WN.

### Discussion

Disturbances of cell cycle control may result in unregulated cell growth and consequently in tumour formation. The cell cycle is governed by a complex network of interacting proteins which at this stage is partially understood (Hartwell and Kastan, 1994). Several major checkpoints have been identified. One of them, the G1 restriction point, is the site at which the decision for cell division is taken. Defects in any of the participating proteins may lead to genomic instability and contribute to the development of malignancy. Mutations and altered expression patterns of genes coding for components of cell cycle regulation have been reported in a variety of human neoplasms (Cordon-Cardo, 1995).

The mutational analysis of metastases from 25 patients with sporadic melanomas in the present investigation shows the occurrence of mutated forms of the *CDKN2B*, *p53* and N-*ras* genes. The same samples had previously been analysed for *CDKN2A* and mutant alleles found (Platz *et al.*, 1996).

The registered alterations comprise point mutations exclusively. No indication of small deletions could be recognised, since all samples efficiently yielded fragments of expected sizes by PCR. Histological examination of the tumour tissue pieces revealed the presence of highly homogeneous tumour cell populations. There was no sign of homozygous deletions involving the investigated gene regions within the limits of the employed PCR method (Platz *et al.*, 1996). The presence of subclones with gene losses may, however, have escaped detection. Altogether, the obtained results show that 10 of 25 patients had metastases with single mutational changes in one of the four investigated genes. One patient had a metastasis which contained both a *CDKN2A* and a *p53* mutation.

The DO-1 monoclonal p53-specific antibody selectively recognised the two p53 missense mutants in the metastases from patients BT and GL but, as expected, did not recognise the truncated protein product in the metastasis

**Table III** Mutations of *CDKN2A*, *CDKN2B*, *p53* and N-*ras* in melanoma metastases

| *Patient* | CDKN2A[a] | CDKN2B[b] | p53[c] | *N*-ras |
|---|---|---|---|---|
| JI | | Pro82Ala CCG/CGG | | |
| MP | | | | |
| Metast.1 | Arg112Gly CGT/GGT | | Arg213Arg CGA/CGG | |
| Metast.2 | Arg112Gly CGT/GGT | | Arg213Arg CGA/CGG | |
| AI | | Asp85Asn GAT/AAT | | |
| BT | | | Pro82Leu CCG/CTG | |
| JA | | | | Gln61Lys CAA/AAA |
| TN | | | Arg213Arg CGA/CGG | |
| HW | C insert codon 4; frame shift and stop at 14 TAG | | | |
| JIR | | | Arg213Arg CGA/CGG | |
| WN | | | | Gln61Arg CAA/CGA |
| EE | His66stop CAC/TAG | | Arg213stop CGA/TGA | |
| GL | | | Val218Gly GTG/GGG | |

[a]Numbering according to Platz *et al.* (1996). [b]Number according to Kamb *et al.* (1994*a*). [c]Numbering according to Buchman *et al.* (1988).

with an exon 6 nonsense mutation from patient EE. All sections from metastases with wild-type p53 showed no immunoreactivity, except for three sections (SB, AI and HW), which showed small subpopulations of immunopositive cells (less than 10% of the cells in 4 $\mu$M sections) and may indicate the presence of p53 mutations. The corresponding frozen tissue pieces used for DNA extraction may contain an even smaller fraction of cells carrying a p53 mutation and might therefore have escaped detection by SSCP and sequence analysis. Stabilisation of the p53 wild-type protein by interaction with other protein components may be an alternative explanation.

To sum up, this investigation registered, among a total of 25 patients, two subjects with an N-*ras* alteration, two with a *CDKN2B* alteration, two with a previously described *CDKN2A* alteration, two with a *p53* and one with both a *CDKN2A* and a *p53* alteration, both resulting in a truncated protein product. Heterogeneous genetic changes may thus contribute to one and the same malignant phenotype. Several additional components of the cell cycle controlling-network may actually be altered in the cases where we could not find any change in the four investigated genes.

The combined results of studies concerning mutations of cell cycle-related proteins in tumours and tumour cell lines reported by many investigators show that such alterations are the most common genetic changes in tumour cells (Clurman and Roberts, 1995). The present mutation screening, while reporting relatively low frequencies of changes for any of the four genes alone, indicates a substantial total frequency of 44% (48% if two silent p53 mutations are counted) of patients with mutations and may hypothetically approach the 100% level when additional genes with functional connection to cell cycle control are included in forthcoming studies.

**Acknowledgements**
This work was supported by grants from the Stockholm Cancer Society, the King Gustaf V Jubilee Foundation, the Ingabritt and Arne Lundberg Research Foundation, the Swedish Radiation Protection Institute and the Swedish Medical Society. The authors wish to express their sincere gratitude to Ulla Hedebrant for excellent technical assistance. We also thank Åsa Lyckhammar and Pia Bülow for secretarial assistance and Marianne Strand for photographic work.

### References

ALBINO AP, NANUS DM, MENTLEV IR, CORDON-CARDO C, MCNUTT NS, BRESSLER J AND ANDREFF M. (1989). Analysis of *ras* oncogenes in malignant melanoma and precursor lesions: correlation of point mutations with differentiation phenotype. *Oncogene*, **4**, 1363–1374.

BALL NJ, YOHN JJ, MORELLI JG, NORRIS DA, GOLITZ LE AND HOEFFLER JP. (1994). Ras mutations in human melanoma: a marker of malignant progression. *J. Invest. Dermatol.*, **102**, 285–290.

BARBACID M. (1978). *Ras* genes. *Annu. Rev. Biochem.*, **56**, 779–827.

BOS JL. (1989). *Ras* oncogenes in human cancer: a review. *Cancer Res.*, **49**, 4682–4689.

BUCHMAN VL, CHUMAKOV PM, NINKINA NN, SAMARINA OP AND GEORGIEV GP. (1988). A variation in the structure of the protein-coding region of the human *p53* gene. *Gene*, **70**, 245–252.

CALDAS C, HAHN SA, DA COSTA LT, REDSTON MS, SCHUTTE M, SEYMOUR AB, WEINSTEIN CL, HRUBAN RH, YEO CJ AND KERN SE. (1994). Frequent somatic mutations and homozygous deletions of the *p16* (MTS1) gene in pancreatic adenocarcinoma. *Nature Genet.*, **8**, 27–32.

CANNON-ALBRIGHT LA, GOLDGAR ED, MEYER LJ, LEWIS CM, ANDERSON DE, FOUNTAIN JW, HEGI ME, WISEMAN RW, PETTY EM, BALE AE, OLOPADE OI, DIAZ MO, KWIATKOWSKI DJ, PIEPKORN MW, ZONE JJ AND SKOLNICK MH. (1992). Assignment of a locus for familial melanoma, MLM, to chromosome 9p13–p22. *Science*, **258**, 1148–1152.

CASTRESANA JS, RUBIO MP, VASQUEZ JJ, IDOATE M, SOBER A, SEIZINGER BR AND BARNHILL RL. (1993). Lack of allelic deletion and point mutations as mechanisms of TP53 activation in human malignant melanoma. *Int. J. Cancer*, **55**, 552–565.

CLURMAN BE AND ROBERTS JM. (1995). Cell cycle and cancer. *J. Natl Cancer Inst.*, **87**, 1499–1501.

CORDON-CARDO C. (1995). Mutation of cell cycle regulators: biological and clinical implications for human neoplasia. *Am. J. Pathol.*, **147**, 545–560.

EL-DIERY DW, TOKINO T, VELCULESCU VE, LEVY DB, PARSONS R, TRENT JM, LIN D, MERCER WE, KINZLER KW AND VOGELSTEIN B. (1993). WAF1, a potential mediator of p53 tumor suppression. *Cell*, **75**, 817–825.

FILMUS J, ROBLES AI, SHI W, WONG MJ, COLOMBO LL AND CONTI CJ. (1994). Induction of cyclin D1 overexpression by activated *ras*. *Oncogene*, **9**, 3627–3633.

FLORENES VA, OYJORD T, HOLM R, SKREDE M, BORRESEN AL, NESLAND JM AND FODSTAF O. (1994). Tp53 allele loss, mutations and expression in malignant melanoma. *Int. J. Cancer*, **69**, 253–259.

GOLDSTEIN A, DRACOPOLI N, ENGELSTEIN M, FRASER MC, CLARK WH AND TUCKER MA. (1994). Linkage of cutaneous malignant melanoma/dysplastic nevi (CMM/DN) to chromosome 9p and evidence for genetic heterogeneity. *Am. J. Hum. Genet.* **54**, 489–496.

GRANA X AND REDDY P. (1995). Cell cycle control in mammalian cells, role of cyclins, cyclin dependent kinases (CDKs), growth suppressor genes and cyclin-dependent kinase inhibitors (CKIs). *Oncogene*, **11**, 211–219.

GRUIS NA, WEAVER-FELDHAUS J, LIU Q, FRYE C, EELES R, ORLOW I, LACOMBE L, PONCE-CASTANEDA V, LIANES P, LATRES E, SKOLNICK M, CORDON-CARDO A AND KAMB A. (1995). Genetic evidence in melanoma and bladder cancers that p16 and p53 function in separate pathways of tumor suppression. *Am. J. Pathol.*, **146**, 1199–1206.

HANNON GJ AND BEACH D. (1994). $p15^{INK4B}$ is a potential effector of TGF-beta induced cell cycle arrest. *Nature*, **371**, 257–261.

HARRIS CC AND HOLLSTEIN M. (1992). p53 tumor suppressor gene. *Principles Pract. Oncol.*, **6**, 1–12.

HARTWELL LH AND KASTAN MB. (1994). Cell cycle control and cancer. *Science*, **266**, 1821–1828.

HEDRUM A, PONTEN F, REN Z, LUNDEBERG J, PONTEN J AND UHLEN M. (1994). Sequence-based analysis of the human p53 gene based on microdissection of tumor biopsy samples. *BioTechniques*, **17**, 118–129.

HOLLSTEIN M, SIDRANSKY Y, VOGELSTEIN B AND HARRIS CC. (1991). p53 mutations in human cancers. *Science*, **253**, 49–53

HUNTER T AND PINES J. (1994). Cyclins and cancer II: cyclin D and CDK inhibitors come of age. *Cell*, **79**, 573–582.

HUSSUSSIAN CJ, STRUEWING JP, GOLDSTEIN AM, HIGGINS PAT, ALLY DS, SHEAHAN MD, CLARK WH, TUCKER MA AND DRACOPOLI NC. (1994). Germline p16 mutations in familial melanoma. *Nature Genet.*, **8**, 15–21.

KAMB A, GRUIS NA, WEAVER-FELDHAUS J, LIU Q, HARSHMAN K, TAVTIGIAN SV, STOCKERT E, DAY III RS, JONSON BE AND SKOLNICK MH. (1994*a*). Cell cycle regulator potentially involved in genesis of many tumor types. *Science*, **264**, 436–440.

KAMB A, SHATTUCK-EIDENS D, EELES R, LIU Q, GRUIS NA, DING W, HUSSEY C, TRAN T, MIKI Y, WEAVER-FELDHAUS J, MCCLURE M, AITKEN JF, ANDERSON DE, BERGMAN W, FRANTS R, GOLDGAR DE, GREEN A, MACLENNAN R, MARTIN NG, MEYER LJ, YOUL P, ZONE JJ, SKOLNICK MH AND CANNON-ALBRIGHT LA. (1994*b*). Analysis of the p16 gene (*CDKN2*) as a candidate for the chromosome 9p melanoma susceptibility locus. *Nature Genet.*, **8**, 22–26.

KISHIMOTO Y, MURAKAMI Y, SHIRAISHI M, HAYASHI K AND SEKIYA T. (1992). Abberations of the p53 tumor suppressor gene in human non-small cell carcinomas of the lung. *Cancer Res.*, **52**, 4799–4804.

LAGERKVIST A, STEWART J, LAGERSTRÖM-FERMER M AND LANDEGREN U. (1994). Manifold sequencing: efficient processing of large sets of sequencing reactions. *Proc. Natl Acad. Sci. USA*, **91**, 2245–2249.

LEVINE AJ, MOMAND J AND FINLEY CA. (1991). The p53 tumor suppressor gene. *Nature*, **351**, 453–456.

MASHIYAMA S, SEKIYA T AND HAYASHI K. (1990). Screening of multiple DNA samples for detection of sequence changes. *Technique*, **2**, 304–306.

MORI T, MIURA K, ACKI T, HISHIHIRA T, MORI S AND NAKAMURA Y. (1994). Frequent somatic mutation of the MTS1/CDK41 (multiple tumor suppressor/cyclin-dependent kinase 4 inhibitor) gene in esophageal squamous cell carcinoma. *Cancer Res.*, **54**, 3396–3397.

NAITO M, SATAKE M, SAKAI E, HIRANO Y, TSUCIDA N, KANZAKI H, ITO Y AND MORI T. (1992). Detection of p53 gene mutations in human ovarian and endometrial cancers by polymerase chain reaction-single strand conformation polymorphism analysis. *Jpn J. Cancer Res.*, **83**, 1030–1036.

NOBURI T, MURA K, WU DJ, LOIS A, TAKABAYASHI K AND CARSON DA. (1994). Detections of the cyclin-dependent kinase-4 inhibitor gene in multiple human cancers. *Nature*, **368**, 753–756.

OHTA M, NAGAI H, SHIMIZU M, RASIO D, BERD D, MASTRANGELO M, SINGH AO, SHIELDS JA, SHIELDS CL, CROCE CL AND HUEBENER K. (1994). Rarity of somatic and germline mutations of the cyclin-dependent kinase 4 inhibitor gene, CDK41, in melanoma. *Cancer Res.*, **54**, 5269–5272.

OKAMOTO A, DEMETRICK DJ, SPILLARE EA, HAGIWARA K, HUSSAIN SP, BENNET WP, FORRESTER K, GERWIN B, SERRANO M, BEACH DH AND HARRIS CC. (1994). Mutations and altered expression of $p16^{INK4}$ in human cancer. *Proc. Natl Acad. Sci. USA*, **91**, 11045–11049.

OTSUKI T, CLARK HM, WELLMAN A, JAFFE ES, RAFFELD M. (1995). Involvement of CDKN2 ($p16^{INK4a}$/MTS1) and $p15^{INK4b}$/MTS2) in pediatric acute lymphoblastic leukemia. *Blood*, **55**, 1436–1440.

PLATZ A, RINGBORG U, MÅNSSON-BRAHME E AND LAGERLÖF B. (1994). Melanoma metastases from patients with hereditary cutaneous malignant melanoma contain a high frequency of N-*ras* activating mutations. *Melanoma Res.*, **4**, 169–177.

PLATZ A, HANSSON J, RINGBORG U, MÅNSSON-BRAHME E, LINDER S, LUNDQVIST E, SEVIGNY P AND INGANÄS M. (1995*a*). Germline p16 mutations are rare in Swedish melanoma families. In *Proceedings of the 38th Annual Clinical Conference*, MD Anderson Cancer Center, Feb 21–24, Houston, TX, pp. 84–85.

PLATZ A, RINGBORG U, GRAFSTRÖM E, HÖÖG A AND LAGERLÖF B. (1995*b*). Immunohistochemical analysis of the N-Ras p21 and the p53 proteins in naevi, primary tumours and metastases of human cutaneous malignant melanoma: increased immunopositivity in hereditary melanoma. *Melanoma Res.*, **5**, 101–106.

PLATZ A, RINGBORG U, LAGERLÖF B, LUNDQVIST E, SEVIGNY P AND INGANÄS M. (1996). Mutational analysis of the *CDKN2* gene in metastases from patients with cutaneous malignant melanoma. *Br. J. Cancer*, **73**, 344–348.

SERRANO M, HANNON JH AND BEACH D. (1993). A new regulatory motif in cell-cycle control causing specific inhibition of cyclin D/CDK4. *Nature*, **366**, 704–707.

SOUSSI T, LEGROS Y, LUBIN R, ORY K AND SCHLICHTHOLZ B. (1994). Multifactorial analysis of p53 alteration in human cancer: a review. *Int. J. Cancer*, **57**, 1–9.

VAN'T VEER JL, BURGERING BMT, VEERSTEG R, BOOT AJM, RUITER DJ, OSANTO S, SCHRIER PJ AND BOS JL. (1989). N-ras mutations in human cutaneous melanoma from sun-exposed body sites. *Mol. Cell. Biol.*, **9**, 3114–3116.

VOLKENANDT M, SCHLEGEL U, NANUS DM AND ALBINO AP. (1991). Mutational analysis of the human p53 in malignant melanoma. *Pigment Cell Res.*, **4**, 35–40.

ZHOU X, TARMIN L, YIN J, JIANG HY, SUZUKI H, RHYU MG, ABRAHAM JM AND MELTZER SJ. (1994). The MTS-1 gene is frequently mutated in primary human-esophageal tumors. *Oncogene*, **9**, 3737–3741.